# Vaginal microbiome variances in sample groups categorized by clinical criteria of bacterial vaginosis

**DOI:** 10.1186/s12864-018-5284-7

**Published:** 2018-12-31

**Authors:** Hui-Mei Chen, Tzu-Hao Chang, Feng-Mao Lin, Chao Liang, Chih-Min Chiu, Tzu-Ling Yang, Ting Yang, Chia-Yen Huang, Yeong-Nan Cheng, Yi-An Chang, Po-Ya Chang, Shun-Long Weng

**Affiliations:** 1 Institute of Bioinformatics and Systems Biology, National Chiao Tung University, Hsinchu, Taiwan; 2 Graduate Institute of Biomedical Informatics, Taipei Medical University, Taipei, Taiwan; 3 Clinical Big Data Research Center, Taipei Medical University Hospital, Taipei Medical University, Taipei, Taiwan; 4 Department of Biological Science and Technology, National Chiao Tung University, Hsinchu, Taiwan; 5 Gynecologic Cancer Center, Department of Obstetrics and Gynecology, Cathay General Hospital, Taipei, Taiwan; 6 Department of Medical Research, Hsinchu MacKay Memorial Hospital, Hsinchu, Taiwan; 7 Department of Medicine, MacKay Medical College, New Taipei City, Taiwan; 8 Department of Obstetrics and Gynecology, Hsinchu MacKay Memorial Hospital, Hsinchu, Taiwan; 9 MacKay Junior College of Medicine, Nursing and Management, Taipei, Taiwan

## Abstract

**Background:**

One of the most common and recurrent vaginal infections is bacterial vaginosis (BV). The diagnosis is based on changes to the “normal” vaginal microbiome; however, the normal microbiome appears to differ according to reproductive status and ethnicity, and even among individuals within these groups. The Amsel criteria and Nugent score test are widely used for diagnosing BV; however, these tests are based on different criteria, and so may indicate distinct changes in the vaginal microbial community. Nevertheless, few studies have compared the results of these test against metagenomics analysis.

**Methods:**

Vaginal flora samples from 77 participants were classified according to the Amsel criteria and Nugent score test. The microbiota composition was analyzed using 16S ribosome RNA gene amplicon sequencing. Bioinformatics analysis and multivariate statistical analysis were used to evaluate the microbial diversity and function.

**Results:**

Only 3 % of the participants diagnosed BV negative using the Amsel criteria (A−) were BV-positive according to the Nugent score test (N+), while over half of the BV-positive patients using the Amsel criteria (A+) were BV-negative according to the Nugent score test (N−). Thirteen genera showed significant differences in distribution among BV status defined by BV tests (e.g., A − N−, A + N− and A + N+). Variations in the four most abundant taxa, *Lactobacillus*, *Gardnerella*, *Prevotella*, and *Escherichia*, were responsible for most of this dissimilarity. Furthermore, vaginal microbial diversity differed significantly among the three groups classified by the Nugent score test (N−, N+, and intermediate flora), but not between the Amsel criteria groups. Numerous predictive microbial functions, such as bacterial chemotaxis and bacterial invasion of epithelial cells, differed significantly among multiple BV test, but not between the A− and A+ groups.

**Conclusions:**

Metagenomics analysis can greatly expand our current understanding of vaginal microbial diversity in health and disease. Metagenomics profiling may also provide more reliable diagnostic criteria for BV testing.

**Electronic supplementary material:**

The online version of this article (10.1186/s12864-018-5284-7) contains supplementary material, which is available to authorized users.

## Background

The human vaginal microbiome has a major influence on women’s health and changes substantially under certain disease conditions. One of the most commonly diagnosed vaginal infections is bacterial vaginosis (BV), which is associated with microbiome composition change and often recurrent. BV is highly prevalent in reproductive-age women, although BV may occur at any age [1]. In addition, approximately half of BV-positive women are asymptomatic [2]. Studies have revealed that BV is associated with a shift in the vaginal flora from *Lactobacillus* predominance to a mixture of organisms, including *Gardnerella vaginalis* and various anaerobic species. However, no single species or combination has been implicated in BV, and the true etiology remains unclear [3].

Two widely used methods for diagnosing BV are the Amsel criteria and Nugent score test. The diagnosis of BV using the Amsel criteria requires the presence of at least three of four markers [4]. In contrast, the Nugent score test is a Gram stain; and its interpretation is based on the assumption that lactobacilli are predominant in healthy women [5]. In addition, both of the methods require experienced medical staff or microscopists to analyze the results. Therefore, the results depend on subjective interpretation.

Sequencing-based research on the human microbiome has seen a marked increase since the Human Microbiome Project was launched by the US National Institute of Health in 2008 [6, 7]. In the past ten years, 16S rRNA gene amplicon sequencing has been widely used in human microbiome research due to its low cost, and time efficiency. Every bacteria has the gene and its conserved regions allow primers to be designed. Thus, it is easy to target a wide variety of bacteria. Although there are strengths and limitations in the 16S rRNA gene amplicon sequencing, it is still a good tool to investigate microbial diversity at genus level [8]. Advances in both sequencing technologies and bioinformatics tools have greatly improved our knowledge of the human vaginal microbiome [9]. In general, these studies have challenged the notion of a ubiquitous “normal” microbiome composition. For example, an early study using culture-independent methods showed that vaginal communities of normal healthy Caucasian women may be dominated by a variety of anaerobic bacteria, aside from *Lactobacillus* species [10]. Furthermore, ethnic groups appear to harbor distinct vaginal microbial communities with different species compositions [11–13].

*Lactobacillus* species are the most abundant vaginal bacteria in the majority of reproductive-age women [13]. A major characteristic of *Lactobacillus* is the production of lactic acid, which may protect against genital tract infections [14–16]. Thus, it is widely believed that a healthy vaginal tract is dominated by *Lactobacillus* species with lower diversity of microbiota. However, not all *Lactobacillus*-deficient vaginal community structures are abnormal or harmful. A recent study found that the vaginal microbiome of most rural Malawi women was dominated by *Gardnerella vaginalis* with limited numbers of *Lactobacillus* spp. after pregnancy [17]. In addition, vaginal *Lactobacillus* abundance is lower in perimenopausal, menopausal, and postmenopausal women compared to reproductive-age women [18, 19]. Culture-independent methods have also revealed three novel uncultivated phylotypes associated with BV [20]. In 2010, eight BV-associated genera were found and validated using quantitative polymerase chain reaction: *Gardnerella*, *Atopobium*, *Megasphaera*, *Eggerthella*, *Aerococcus*, *Leptotrichia/Sneathia*, *Prevotella*, and *Papillibacter* [21]. Subsequently, a dynamic study identified recurrence treatment failure in BV using deep sequencing analysis [22].

Due to ethnic variance and the high relapse rate of BV after treatment, the present study focused on female Taiwanese adults suspected to have BV to examine variation in the microbiome according to the Amsel criteria and Nugent score test classification. In addition to identifying BV-associated microbiota, we were interested in understanding further the association between the microbial ecology of the vaginal community and individual BV test results.

## Methods

### Sample collection

Women were recruited at the Department of Obstetrics and Gynecology at the Mackay Memorial Hospital (Hsinchu, Taiwan). None of the women were pregnant, all had a history of sexual activity, and none had taken any antibiotics or vaginal antimicrobials (orally or by topical application to the vulvar/vaginal area) within the preceding 2 months. The women had not engaged in sexual activity in the 48 h before the visit. Women were excluded if they had used douches, had an active infection with *Chlamydia* spp., yeast, *Neisseria gonorrhoeae*, or *Trichomonas vaginalis*, had a history of diabetes mellitus, were fitted with an IUD. Women volunteered and gave written informed consent to participate in the study.

Vaginal samples were collected at the initial visit. Two vaginal swabs were placed on the mid-vaginal wall. The first swabs used were from an ESwab™ Collection Kit 480C (Copan Diagnostics, Inc., Murrieta, CA, USA), and they were stored at 4 °C within 4 h. They were kept at − 80 °C for long-term storage but for no longer than 6 months. The second swabs used were cotton-tipped swabs. The swabs were rolled by a clinical physician on to a microscope glass slide that was air-dried, Gram-stained, and then scored using the Nugent score test.

### Clinical criteria of bacterial vaginosis

The diagnostic tests for BV used were the Amsel criteria and Nugent score test [4, 5]. According to the Amsel criteria, patients with BV must present with three of the four following criteria: (1) an elevated vaginal pH of > 4.5; (2) a homogeneous, white or gray “non-inflammatory” discharge that smoothly coats the vaginal walls; (3) a fishy smell to this discharge before or after the addition of 10% potassium hydroxide; and (4) the presence of “clue cells” (squamous epithelial cells covered with adherent bacteria) on microscopic examination.

The Nugent score reflects the relative abundances of three kinds of bacterial cell morphotypes in Gram-stained vaginal smears: large, Gram-positive rods (*Lactobacillus* morphotypes); small, Gram-variable rods and cocci (*G. vaginalis*, *Porphyromonas*, and *Peptostreptococcus* morphotypes); and curved, Gram-variable rods (*Mobiluncus* spp. morphotypes). The Nugent scores range from 0 to 10, with 0–3 considered normal, 4–6 intermediate, and 7–10 indicative of BV. Vaginal pH was measured using pH-Fix test strips (MACHEREY-NAGEL GmbH & Co. KG, Dueren, Germany), and scored by a clinical physician according to the manufacturer’s instructions with a scale ranging from 4.0–7.7.

### DNA extraction

A QIAamp DNA Blood Mini Kit (Qiagen, Hilden, Germany) was used for DNA extraction. The procedure was performed following the manufacturer’s protocol with minor modifications. Briefly, each sample was centrifuged at 13,000 rpm for 2 min, and the resulting bacterial pellet was resuspended in 180 μl of enzyme solution (20 mg/ml lysozyme, 20 mM Tris-HCl [pH 8.0], 2 mM ethylenediaminetetraacetic acid, and 2% sodium dodecyl sulfate). Lysates were incubated at 37 °C for 30 min prior to the addition of 20 μl proteinase K (25 mg/ml) and 200 μl Buffer AL. Each suspension was subsequently incubated at 56 °C for 30 min, and for a further 15 min at 95 °C. The final DNA was eluted with 30 μl of Buffer AE, and stored at − 20 °C for further analysis.

### Library construction and sequencing of the V4 region of the 16S ribosomal RNA

The PCR primers F515 (5’-GTGCCAGCMGCCGCGGTAA-3′) and R806 (5’-GGACTACHVGGGTWTCTAAT-3′) were designed to amplify the V4 region of the bacterial 16S rDNA as described in a previous study [23]. PCR amplification was performed in a 50-μl reaction volume containing 25 μl 2× Phusion® Flash PCR Master Mix (Thermo Fisher Scientific, Waltham, MA, USA), 0.5 μM of each forward and reverse primer, and 50 ng of DNA template. The reaction process increased the initial temperature to 98 °C for 30 s, followed by 30 cycles of 98 °C for 10 s, 54 °C for 30 s, 72 °C for 30 s, and a final extension step of 72 °C for 5 min. Next, the amplified products were checked by 2% agarose gel electrophoresis. Amplicons were purified using the AMPure® XP PCR Purification Kit (Agencourt Bioscience Corp., Beverly, MA, USA), and quantified using a Qubit™ dsDNA HS Assay Kit (Thermo Fisher Scientific) on a Qubit® 2.0 Fluorometer (Thermo Fisher Scientific), all according to the respective manufacturers’ instructions. For V4 library preparation, Illumina adapters (Illumina, San Diego, CA, USA) were attached to the amplicons using an Illumina TruSeq DNA Sample Preparation v2 Kit (Illumina). Purified libraries were used for cluster generation and sequencing with 2 × 150 reagent using the MiSeq system (Illumina).

### Filtering 16S rRNA sequencing data for quality

Sequencing reads from different samples were identified and separated according to specific barcodes at the 5′ of the sequence. The FASTX-Toolkit (http://hannonlab.cshl.edu/fastx_toolkit) was employed to process the raw read data files, and the reads were processed for sample identification and trimming barcode, adaptor, and low quality bases. The bases with quality lower than Phred quality score 20 were removed. Sequences consisting of less than 100 nucleotides were discarded along with any reads containing ambiguous characters.

### Taxonomy assignment for bacteria 16S rRNA sequence

The 16S rRNA gene sequences were collected from the NCBI nucleotide collection database. There were 552,528 distinct 16S rRNA bacteria reference sequences. To classify the microbiome of the vagina, Bowtie2 [24], a fast and memory efficient aligning sequencing reads tool, was adopted with the parameter “very sensitive” to map the reads to bacterial 16S rDNA sequences. The paired-end quality filtering reads were aligned to reference sequences. The paired-end reads that mapped to specific bacteria with a sequence similarity exceeding 97% were included in operational taxonomic unit (OTU) table. A total of 4,045,760 aligned sequencing reads were obtained, and on average, 52,542 sequencing reads were identified per vaginal subject. We sorted the sequences into 1349 operational taxonomic units (OTUs, ≥ 97% identity of 16S rRNA gene).

### Statistical analysis

The OTU table of raw counts was normalized to an OTU table of relative abundances, and taxa of the same type were agglomerated at the phylum, class, order, family, and genus level via the Greengenes database [25–27]. Biodiversity was compared between classified groups using the nonparametric Wilcoxon test. Pearson correlation coefficient were used to test the association between richness (species number) and Shannon diversity index. To investigate how these bacteria were related, we first calculated the average sum of relative abundance for each genus in the whole sample, and used principal component analysis for the 20 most abundant genera. Phylogeny based weighted UniFrac analysis was also performed [28]. The one-way analysis of similarity (ANOSIM) and the analysis of similarity percentage (SIMPER) were used to investigate the differences of vaginal microbiota across group. With clustering and principal coordinate analysis, the pattern of genera in the classified group were observed. Based on the characteristics of the compositional data, a correlation network of specific genera was built using SparCC correlation coefficients [29]. R programming software was used for the statistical analyses (The R Project for Statistical Computing, Vienna, Austria). PAST software (Paleontological Statistics) was used to evaluate one-way analysis of similarity (ANOSIM) and the analysis of similarity percentage (SIMPER). PICRUSt was used to analyze the potential functions of bacterial communities [30].

## Results

### Characteristics of the study population

A total of 77 women (median age, 41 years) were enrolled in this study. They were diagnosed using both the Amsel criteria and Nugent score test. For convenience, the symbol A+ is used to indicate the female patients meeting three of the four Amsel criteria and A− to indicate all the others. Similarly, three symbols are used to represent the Nugent score test: N+ for patients with score of > 7.0, indicating BV infection, N* for those with scores between 4.0 and 6.0, indicating “intermediate flora” and N− for those with score < 3.0, indicating normal flora.

**Fig. 1:**
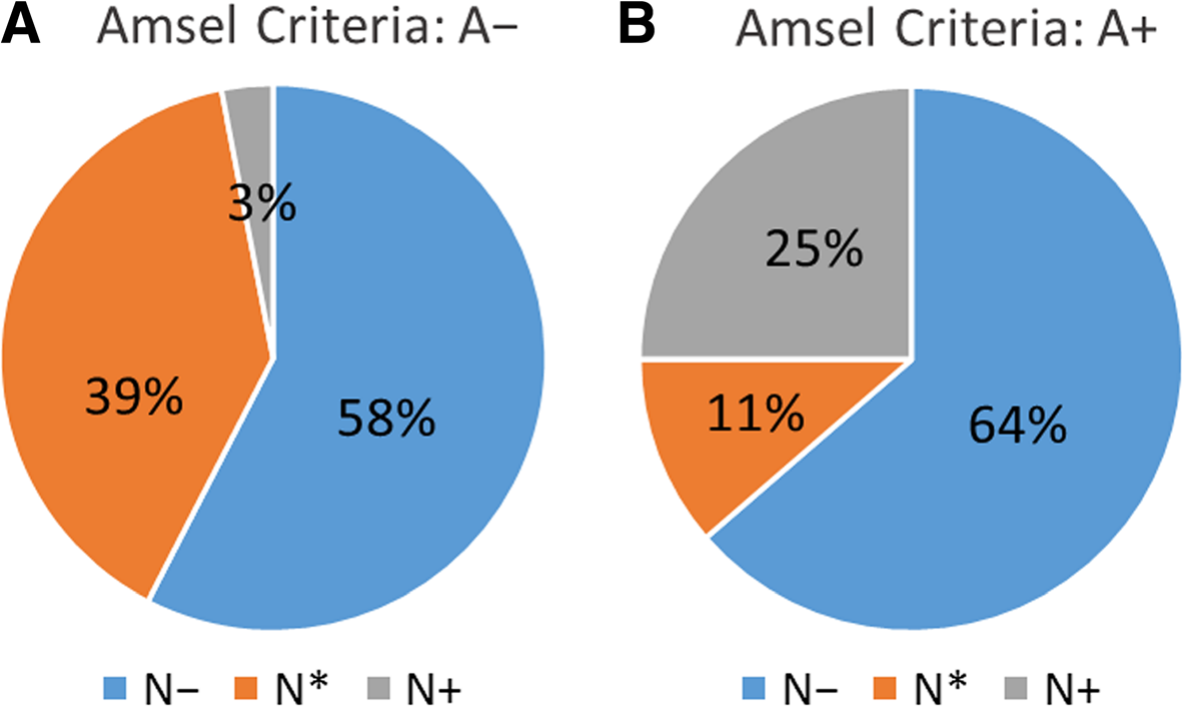
Proportions of individuals with different Nugent score test results (N−, N*, N+) within Amsel criteria groups. **a** In the A− group, only 3% of women were diagnosed BV-positive (N+) using the Nugent scoring test. **b** In the A+ group, up to 64% of women were diagnosed BV-negative (N−) using the Nugent scoring test

Agreement between these tests was surprisingly weak. 3% classified as the A− group were BV-positive (N+) according to the Nugent score test, whereas 64% of the women in the A+ group had “normal flora” (N−) (Fig. 1). Participants were further classified into the following six groups based on the two BV tests: A − N−, A − N*, A − N+, A + N−, A + N*, and A + N+ (Table 1**)**. Since one participant only was in the A − N+ group, this group was excluded from comparative analysis (so we refer to five groups in most instances).

**Table 1:** Descriptive characteristics of the study population

Groups		Age					pH Value			
	N	Min.	Mean	Max.	SD	SE	4.5	5.0	5.0–6.0	6.5+
A − N−	19	22	41	62	10.42	2.46	6	4	4	5
A – N*	13	49	61	83	10.11	2.80	2	0	1	10
A − N+	1	53	53	53	NA	NA	0	0	1	0
A + N−	28	21	34	59	7.83	1.48	0	22	5	1
A + N*	5	26	44	74	19.73	8.82	0	2	1	2
A + N+	11	21	31	41	6.49	1.96	0	5	6	0
Total	77						8	33	18	18

* Amsel test: A− means bacterial vaginosis (BV)-negative; A+ means BV-positive

* Nugent score: N− means BV-negative; N* means intermediate; N+ means BV-positive

* *Abbreviations*: *SD* Standard deviation, *SE* Standard error

### Four dominant phyla in the vaginal bacterial communities

**Fig. 2:**
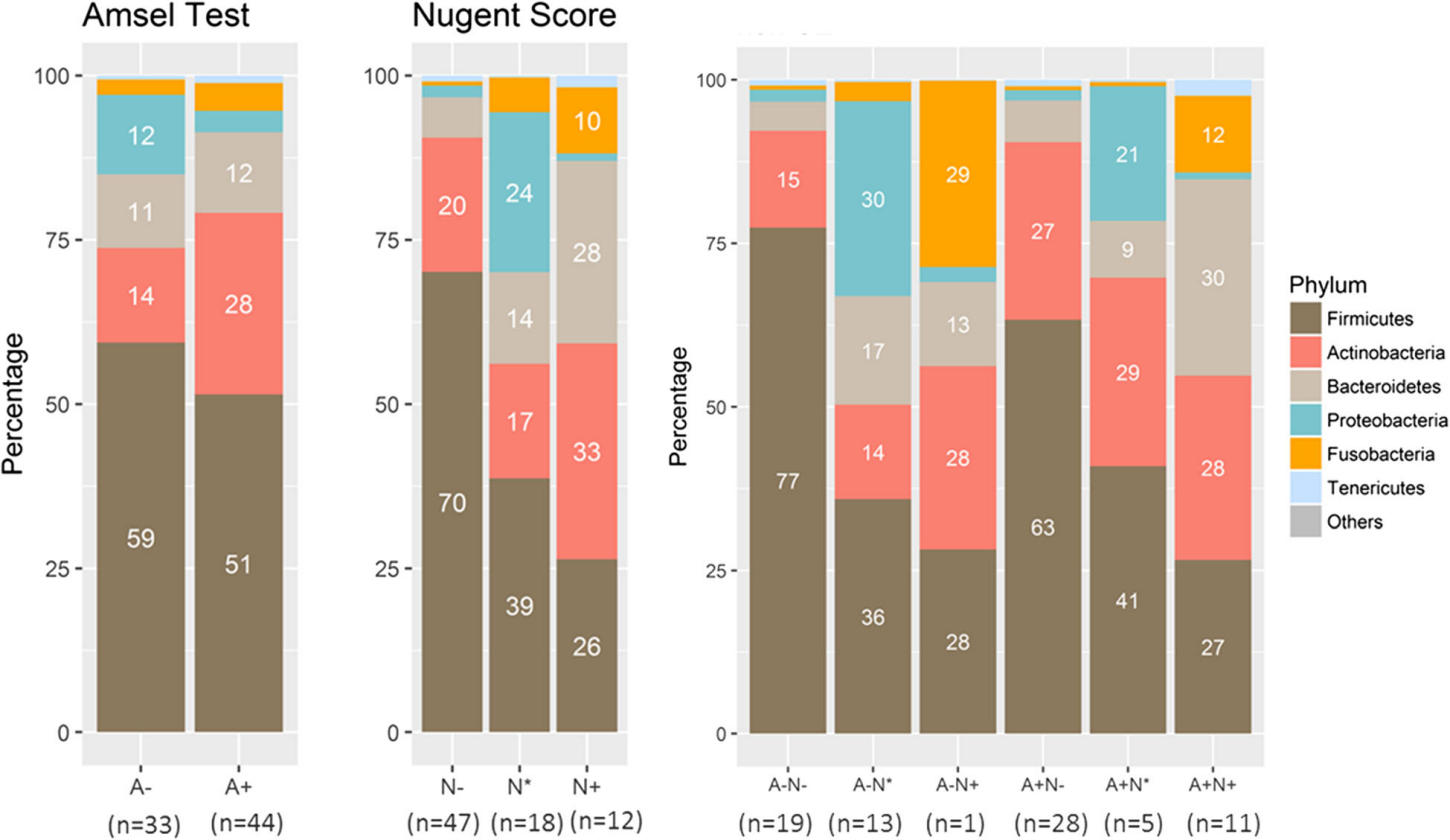
Vaginal microbiota composition at phylum level. Firmicutes was the most dominant phyla. Composition differed only lightly between the A− and A+ groups, but was much higher in the N− group than the N* and N+ groups. The microbiota composition in the A − N– group was less complex than the other groups

Four phyla, Firmicutes, Actinobacteria, Bacteroidetes, and Proteobacteria, were detectable in all the vaginal samples; and Fusobacteria and Tenericutes were present in most of them (Additional file 1: Figure S1). Figure 2 depicts the average relative abundance of six phyla (Firmicutes, Actinobacteria, Bacteroidetes, Proteobacteria, Fusobacteria, and Tenericutes) for each BV test group. Firmicutes relative abundance differed little between the A− and A+ groups (59% vs. 51%) but was much higher in the N− group than in either the N* or N+ groups (70% vs. 39 and 26%). Relative abundance of Actinobacteria, Bacteroidetes, and Proteobacteria were similar in the A− group but differed significantly in the N− group. Thus, the same status on the Amsel criteria and Nugent test was not indicative of similar microbiome condition. Additionally, Actinobacteria in the A− group was much lower than the A+ group (14% vs. 28%). Remarkably, Proteobacteria was higher in the N* group (24%) than in either the N− group or N+ group.

The most abundant phyla within each group differed substantially among the six BV test groups. Firmicutes was dominant in the four groups: A − N− (77%), A + N− (63%), A + N* (41%), and A − N* (36%), while Firmicutes and Proteobacteria were most abundant in the A − N* group (36 and 30%, respectively). In the A + N+ group, the abundance of three phyla, Firmicutes, Actinobacteria, and Bacteroidetes, were similar and collectively accounted for to 85% of the microbiota. In the A − N+ group, Firmicutes, Actinobacteria, and Fusobacteria each accounted for approximately 30%; however, there was only one such vaginal sample.

### Variations in alpha diversity of the vaginal microbiota

**Fig. 3:**
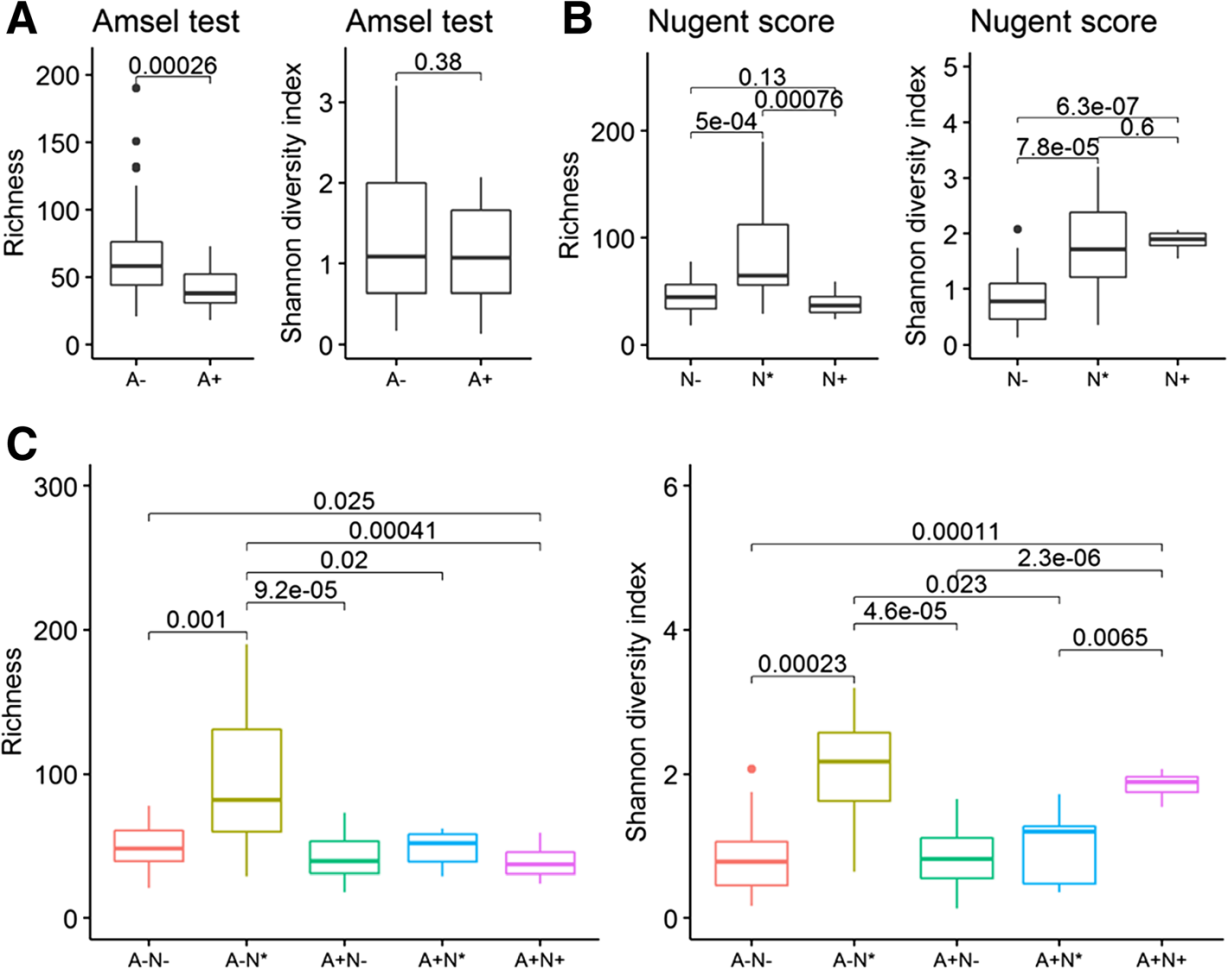
Box plots for species richness and Shannon index diversity at the genus level. **a** Amsel criteria. Richness differed significantly between the Amsel groups (A+ and A−). **b** Nugent score test. Both richness and Shannon diversity index generally differed markedly for all pair-wise comparisons among Nugent test score groups (N−, N*, and N+). **c** The five groups defined by both the Amsel criteria and Nugent score test. The richness of the vaginal community was significantly higher in the A − N* group compared to all other groups

On average, 53 genera were identified per subject and 353 genera were detected in total. In this study, the richness of microbial diversity defined as the number of genera detected within each subject. Richness differed significantly between the Amsel groups (A+ and A−); however, these groups did not differ in Shannon diversity index (Fig. 3a). In contrast, both richness and Shannon diversity index generally differed markedly for all pair-wise comparisons among Nugent test score groups (N−, N*, and N+) (Fig. 3b). Detailed descriptive statistics of the Shannon diversity index and richness for all groups are present in Additional file 2: Table S1 and Table S2, respectively.

The Pearson correlation coefficient between the richness and Shannon diversity index was weakly positive in the A+ group (*r* = 0.12) but strongly positive in the A− group (*r* = 0.81) (Additional file 3: Figure S2A). The correlation was strongly positive in the N* group (*r* = 0.86) and of intermediate strength in the N− and N+ groups (both around 0.4) (Additional file 3: Figure S2B). Among the dive groups defined by both BV test, the A − N* group showed the strongest correlation (*r* = 0.89) (Additional file 3: Figure S2C).

There were significant differences in microbial diversity among the five groups classified by the two BV tests (*P* < 0.05, Fig. 3c). Remarkably, the richness of the vaginal community was significantly higher in the A − N* group compared to all other groups. However, this group mainly consists of elderly women (mean age of 61 years, Table 1). Furthermore, richness did not differ significantly among the A + N−, A + N*, and A + N+ groups. In contrast, the Shannon diversity index differed among these groups, being higher in the A + N+ group than the A + N− and A + N* groups. In addition, we examined these relationships in two A − N− subgroups separated by age (21–41 and 46–62 years old). Neither richness nor the Shannon diversity index differed between these subgroups (Additional file 4: Figure S3).

### Beta diversity in vaginal community with different detection results

The variance of β-diversity among individuals was evaluated using two methods, the UniFrac distance [28] and Bray-Curtis similarity distance. As Table 2 shows, the one-way analysis of similarity (ANOSIM) results indicated that the Amsel criteria groups (A– and A+) did not differ significantly, and the R values for these groups were near zero using either the UniFrac metric or Bray-Curtis metric. In contrast, the Nugent score test yielded better group discrimination based on the weighted UniFrac distance or Bray-Curtis distance (*R* > 0.4, *P* = 0.0001). As Table 3 shows, ANOSIM percentage (SIMPER) results using the Bray-Curtis distance yielded on overall average dissimilarity of 62.11% between the Amset criteria groups, substantially lower than among the Nugent score test groups (71.45% for N– vs. N*, 69.36% for N* vs. N+, and 75.01% for N– vs. N+, respectively). Across all the groups, the four most abundant taxa responsible for dissimilarity were *Lactobacillus*, *Gardnerella*, *Prevotella*, and *Escherichia*; however, the rank order differed among individual groups. *Lactobacillus* made the greatest contribution to dissimilarity between all group pairs (32.91, 35.67, and 36.74%) except for the comparison between in the N* and N+ groups (12.55%), for which the greatest dissimilarity was generated by *Escherichia* (16%) followed by *Prevotella* (approximately 15%).

**Table 2:** Results from Analysis of Similarities (ANOSIM) between groups (UniFrac distance and Bray–Curtis similarity measurements)

Comparisons	Unweighted UniFrac Distance		Weighted UniFrac Distance		Bray–Curtis	
	R	*P*-value	R	*P*-value	R	*P*-value
A− vs. A+	0.1263	0.0004	0.0033	0.3426	0.0262	0.1193
N− vs. N*	0.1290	0.0771	0.4030	0.0003	0.4006	0.0003
N− vs. N+	−0.0105	1.0000	0.4077	0.0003	0.4970	0.0006
N* vs. N+	−0.1516	1.0000	0.3828	0.0003	0.2155	0.0102
Global effect	*R* = 0.08503, *p* = 0.0677		*R* = 0.4624, *p* = 0.0001		*R* = 0.4266, p = 0.0001	

* The *P*-values for Bonferroni correction

**Table 3:** Results of SIMPER analysis (Bray–Curtis similarity measurements)

Overall Average Dissimilarity	A− vs. A+		N− vs. N*		N* vs. N+		N− vs. N+	
	62.11%		71.45%		69.36%		75.01%	
Contribution %	*Lactobacillus*	32.91	*Lactobacillus*	35.67	*Escherichia*	16.28	*Lactobacillus*	36.74
	*Gardnerella*	12.98	*Escherichia*	15.78	*Prevotella*	14.95	*Prevotella*	16.01
	*Prevotella*	11.28	*Gardnerella*	10.69	*Lactobacillus*	12.55	*Gardnerella*	11.26
	*Escherichia*	9.49	*Prevotella*	9.34	*Gardnerella*	10.72	*Sneathia*	8.52

Principal coordinate analysis plots revealed no major break among groups (Additional file 5: Figure S4). However, 74% of the subjects in the N− group (35/47 = 0.7446), 50% in the N* group (9/18 = 0.5), and 75% o in the N+ group (9/12 = 0.75) were clustered together according to hierarchical clustering using Bray-Curtis distance (Additional file 6: Figure S5). Thus, some microbiome clusters were highly correlated with the Nugent score test groups class.

### Differences in specific genera among sample groups

**Fig. 4:**
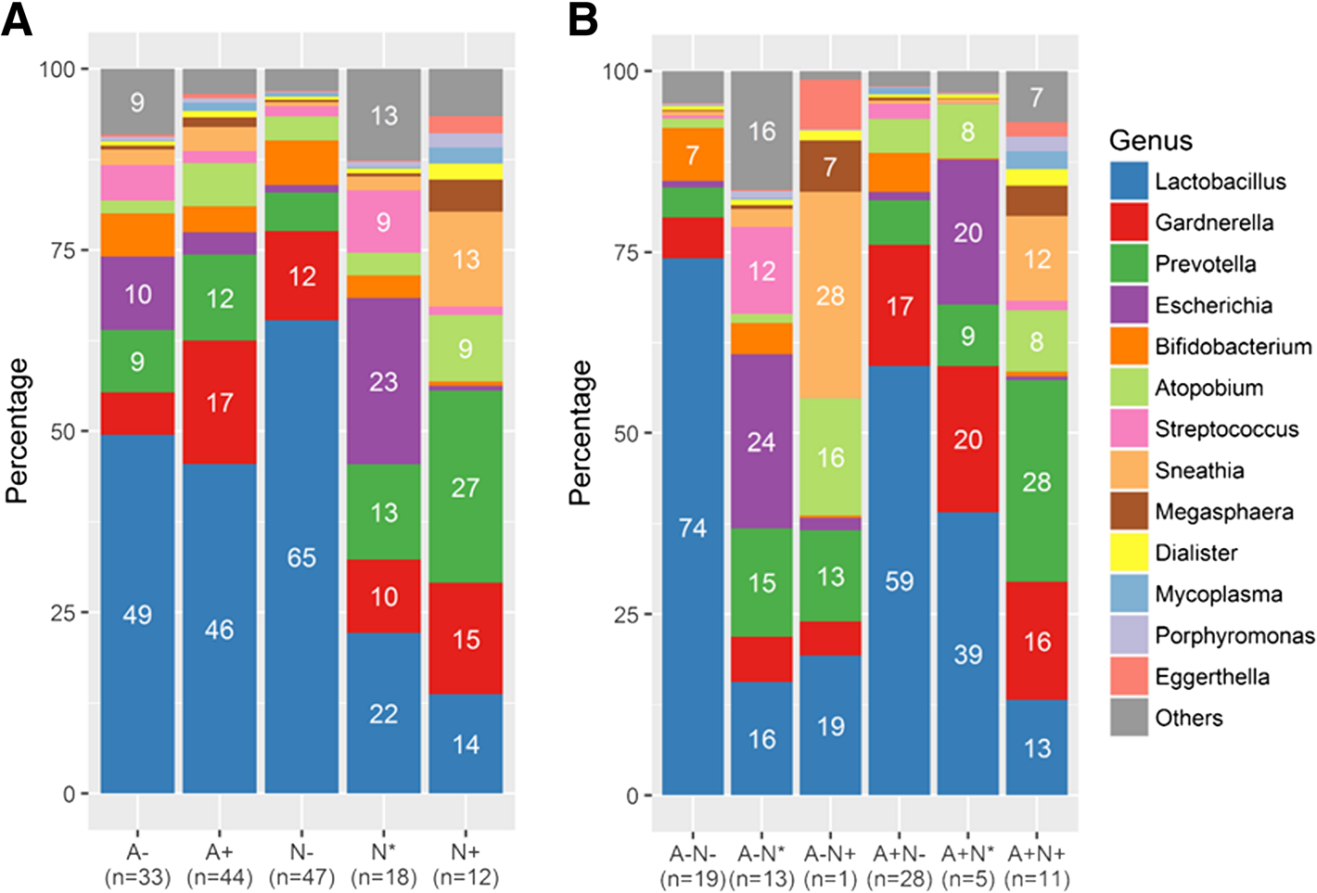
Specific genera differed markedly within groups. **a** and **b** are bar charts for average sum of relative abundance at genus level within groups. **a** Lactobacillus abundace was the highest in the N– group, but it was similar in the A– and A+ groups. **b** The microbial diversity was more eveness in the A–N* and A+N+ groups than in the other groups

Figure 4 presents the distributions of the 13 most abundant vaginal genera among the groups. The relative abundance of *Lactobacillus* species was highest in the N− group (65%), whereas *Lactobacillus* species abundance was similar in the A− and A+ groups (49 and 46%, respectively) (Fig. 4a). In the N+ group, *Prevotella* was the most abundant genus (27%), not *Gardnerella* (15%), whilr *Escherichia* was the most abundant genus in the N* group (23%) (Fig. 4a).

**Fig. 5:**
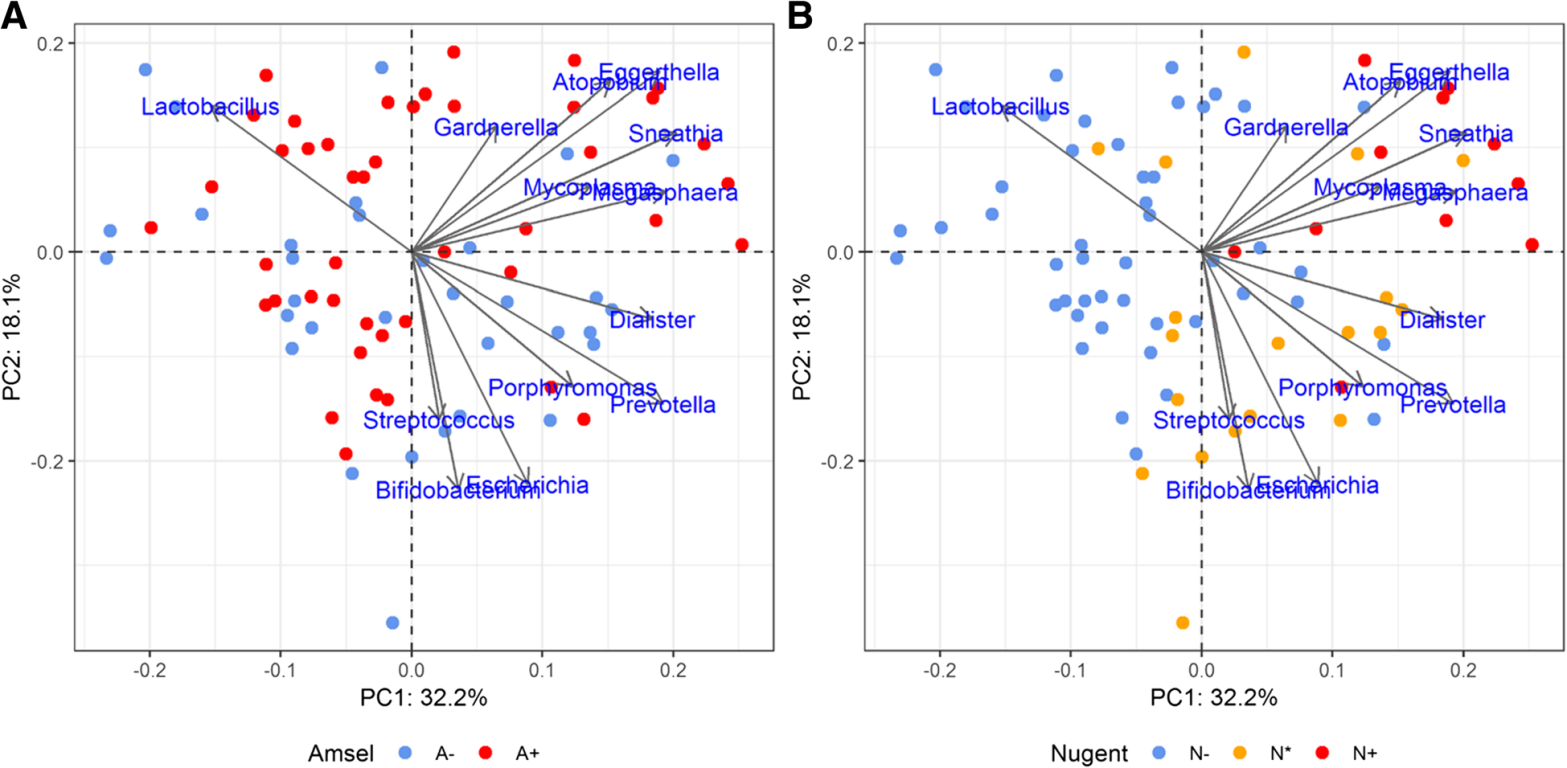
Principal component analysis (log2-transformed) of the 13 most abundant genera. The first two components are plotted (PC1: 32.2% variance; PC2: 18.1% variance). Individuals are represented by points. The length of the vectors represents the effect of each component on the status. **a** The two colors represent the test resutls of the Amsel criteria. Red color means A+, and blue color means A–. **b** The three colors represent the three test restuls of the Nugent score test. Red color means N+, yellow color means N*, and blue color means N–.

*Lactobacillus* species dominated most vaginal communities in the A − N− group (74%) and A + N− group (59%), whereas the A − N* and A + N+ groups exhibited more heterogeneous dominance, including samples with *Lactobacillus*, *Gardnerella*, *Prevotella*, *Streptococcus*, *Atopobium*, or *Sneathia* dominance (Fig. 4b). As Fig. 5 shows, principal component analysis revealed generally negative correlations between *Lactobacillus* and all other genera, while the proportions of these other genera were mainly positively correlated, including a strong correlation between *Bifidobacterium* and *Streptococcus*.

The distribution of most abundant genera in all vaginal samples were presented in Additional file 7: Figure S6. The relative abundance of *Lactobacillus* species was highly variable among individuals within groups: 5%–97% in A − N−, 0.4–29% in A − N*, 1.1–98% in A + N−, 2.5–92% in A + N*, and 1.1–31% in A + N+. Similarly, the relative abundance of *Gardnerlla* among individuals ranged from 0.5–32.5% in A − N−, 0.03–34.6% in A − N*, 0.05–75% in A + N−, 0.5–49.9% in A + N*, and 3–45.4% in A + N+.

### Correlation networks and functional analysis of vaginal microbiome groups

**Fig. 6:**
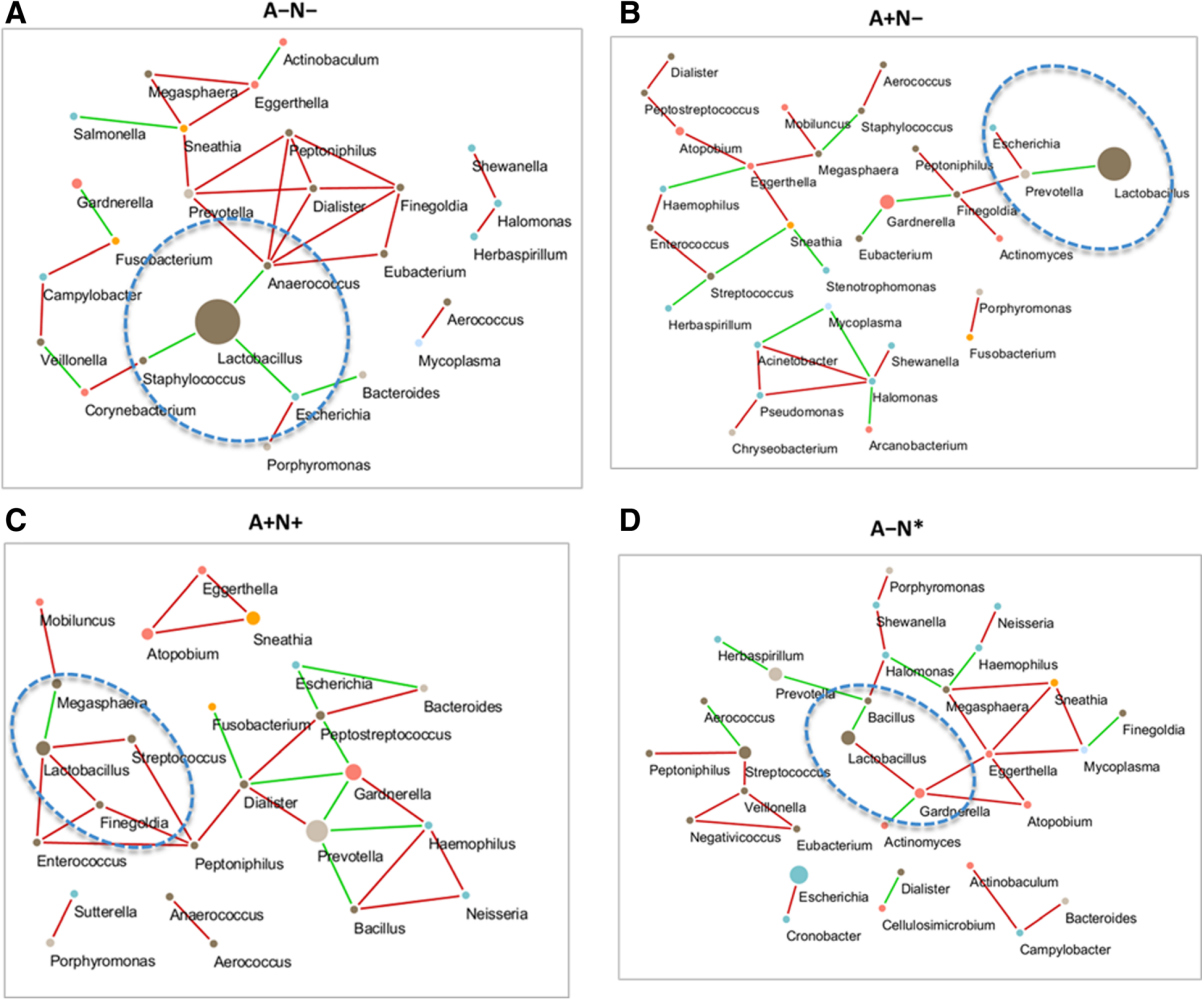
Correlation network of vaginal microbiota within groups. The figures show correctional networks using SparCC correlation coefficients at the genus level. The nodes represent genera, and the node size represents the relative abundance. An edge is colored green for a negative correlation and red for a positive correlation. **a** A − N− group. **b** A + N+ group. **c** A + N− group. **d** A − N* group

Microbial correlation networks were built using SparCC [29]. Seventy-four genera were selected based on the following two conditions: 1) present in at least 50% of subjects within one subgroup; and 2) differing significantly between at least two groups (*P* < 0.05). Network nodes were defined when correlation coefficients were among the top 30 absolute values for each group. In Fig. 6, nodes with the same color indicate the same phylum.

In the A − N− group, *Lactobacillus* was negatively correlated with *Staphylococcus*, *Escherichia*, and *Anaerococcus* (Fig. 6a), while in the A + N− group*, Lactobacillus* was negatively correlated with *Prevotella* (Fig. 6b). In the A + N+ group, *Gardnerella* showed a strong negative correlation with *Peptostreptococcus*, *Dialister*, and *Prevotella* (Fig. 6c), whereas in the A − N* group, *Gardnerella* was negatively correlated with *Actinomyces* and positively correlated with *Lactobacillus* (Fig. 6d). In the A + N* group, *Lactobacillus* was negatively correlated with *Escherichia* and positively correlated with both *Staphylococcus* and *Pseuodomonas* (Additional file 8: Figure S7).

Functional profiling of microbial communities was conducted according to the KEGG database using PICRUSt. As Table 4 shows, at level three of the KEGG database, 152 predictive functional categories differed significantly between the A − N− and the A − N* groups (*P* < 0.05), and 90 predictive functional categories differed between the A − N− and A + N+ group (*P* < 0.05).

**Table 4:** Number of predictive functional categories

	*P*-value < 0.05	adj.*P* < 0.05
A − N− vs. A − N*	152	14
A − N− vs. A + N+	90	20
A − N− vs. A + N−	0	0
A − N− vs. A + N*	15	0
A + N− vs. A + N*	2	0
A + N− vs. A + N+	70	7
A + N* vs. A + N+	7	0

* *P*-values are evaluated by Wilcoxon rank sum test

* adj.P: The *P*-values for Bonferroni correction

Some predictive functional categories differed significantly between groups (Additional file 9: Table S3-S8). However, there was no significant difference in any of the predictive functional categories between the A − N− group and A + N− group. Thirty-two predictive functional categories differed significantly between any two of the five BV test groups, including bacterial chemotaxis and bacterial invasion of epithelial cells (Additional file 9: Table S9). Using the principal component analysis of these functional categories, the vaginal subjects were separated into two clusters corresponding to the Nugent score test. One cluster consisted mostly of N– subjects, and the other cluster consisted of N* and N+ subjects with few N– subjects (Additional file 10: Figure S8). Besides, there are 12 of the 32 functional categories that differed significantly among the Nugent score test groups but not the Amsel criteria groups (Additional file 11: Figure S9).

## Discussion

While the clinical symptoms associated with BV are relatively easily measured, it is difficult to have a consistent detection method to verity the cause of BV. According to the literature, the clinical syndrome of BV is most likely caused by disrupted or dysbiotic vaginal microbiota. The major purpose of this study was to investigate the relationships between the vaginal microbiome composition and BV status according to the results of the Amsel criteria and Nugent score test.

In our study, the results of the Amsel criteria and Nugent score test were poorly consistent. Only 25% of participants in the A+ group were in the N+ group (Fig. 1). One possible reason for this inconsistency is the inclusion of both reproductive-age and postmenopausal women, resulting in a less focused and unfavorably wide age range within participants. Another possible reason may be the subjective judgements of the different technicians assigning the Nugent scores, or of the physicians assigning the Amsel criteria. The participants included reproductive-age and postmenopausal women even though there is still no adequate method for BV diagnosis in peri- and post-menopausal women [31, 32]. Although the Nugent score test was originally designed and validated for pregnant women, it is the standard laboratory test used in Taiwan for the diagnosis of BV [5]. According to the vaginal environments of the test results, we can observe the major differences of vaginal microbiome in these various vaginal communities, while at the same time we can evaluate how the vaginal ecology corresponds to the test results.

In our study, the major differences between the A − N− and A + N− groups are the distribution of vaginal pH values, *Lactobacillus* abundance, and *Gardnerella* abundance. No participants in the A + N− group had vaginal pH < 4.5. In contrast, nearly one-third of participants in the A − N− group had vaginal pH < 4.5 (Table 1). Earlier studies have shown that BV is initiated by the sexual transmission of *Gardnerlla vaginalis*, which is a Gram-variable-staining facultative anaerobic bacteria [33]. However, *G. vaginalis* is also a normal microbiota in healthy women [34, 35]. In our study, 40% of the vaginal samples in the A + N− group had less than 50% *Lactobacillus* abundance with noticeable *Gardnerella*, *Prevotella*, or *Atopobium*. In contrast, all vaginal samples in A − N− group were dominated by *Lactobacillus* or *Bifidobacterium* with little *Gardnerella* abundance (Additional file7: Figure S6). These results are consistent with the previous studies [13].

Vaginal pH and microbiome composition differed between reproductive-age and post-menopausal women [36, 37]. In this study, the minimum age in the A − N* group was 49 years, and most of the participants in this group had vaginal pH > 6.0. Moreover, the biodiversity differed significantly between the A − N* group and all other BV test groups (Fig. 3c). It could be that the vaginal microbiota in the A − N* group is undergoing a shift from being dominated by lactic acid bacteria to more diverse anaerobic species [38, 39]. Therefore, the participants in the A − N* group could be at high risk of aerobic vaginitis due to the markedly higher proportion of *Escherichia*, and *Streptococcus* than in the other groups [40, 41].

In addition to *G. vaginalis*, *Prevotella* spp. and *Atopobium* spp. have been found to be associated with BV using culture methods and quantitative PCR assessments [42, 43]. In our study, participants in the A + N+ group were between 21 and 41 years old. Each vaginal sample was dominated by *Prevotella, Gardnerlla*, *Atopobium*, or *Sneathia*, and had low *Lactobacillus* abundance. It is consistent with previous studies. In addition, bacterial chemotaxis and bacterial motility influence host infection and pathogenicity [44, 45]. In our study, the predictive functional expressions of bacterial chemotaxis and bacterial motility in the A + N+ group were significantly higher than in the A − N− group. Therefore, a variety of vaginal microbiota exhibiting chemotaxis and motility may play important roles in the survival of some bacteria in the vaginal community.

Current hypotheses of pathogenesis of BV include racial and societal differences, intravaginal practices, sexually transmitted infections and human immunodeficiency virus [46–49]. The major aim of this study was to examine variations in vaginal microbiome among women with suspected BV. However, we could not determine which genera actually resulted in BV. Such an analysis is limited by the heterogeneity of microbiome profiles among subjects due to the inclusion of both reproductive-age and postmenopausal women. In addition, hormonal changes, such as those during the menstrual cycle, are major factors influencing vaginal microbiota composition. Thus, for studies on causative pathogens, it may be advantageous to focus microbiome profiling specifically on reproductive-age, perimenopausal, or postmenopausal women individually. Second, it would be valuable to examine the variation in vaginal microbiota over time to gain a clearer understanding both of the individual species function and pathogenicity.

## Conclusions

In this study, we examined the microbial composition of vaginal samples using culture-independent methods according to the classification by the Amsel criteria and Nugent score test for BV. We demonstrated marked inconsistency between tests and heterogeneity in microbiome profiles both among BV test groups and within groups. This heterogeneity may arise in part from the inherent differences between reproductive-age and postmenopausal women, but also from the subjective nature of these tests, and individual differences among the women tested. Nonetheless, these results provide a foundation for improved diagnostics, health promotion, and individualized treatment.

## Additional files

Additional file 1: **Figure S1.** Phylum-level microbiota of vaginal samples. (PDF 222 kb)

Additional file 2: **Table S1**. Descriptive statistics of Shannon diversity and richness. **Table S2**. The *p*-values for any two groups (PDF 282 kb)

Additional file 3: **Figure S2.** The Pearson correlation coefficients between richness and Shannon diversity index. (PDF 405 kb)

Additional file 4: **Figure S3.** Boxplot of richness and Shannon diversity index in the two subgroups of the A − N− group. (PDF 206 kb)

Additional file 5: **Figure S4.** Principal coordinate analysis (PCoA) plots of vaginal bacterial communities. The PCoA plots were generated by the unweighted UniFrac distance, weighted UniFrac distance, and Bray-Curtis distance. (PDF 426 kb)

Additional file 6: **Figure S5.** Hierarchical clustering method with Bray-Curtis metric. (PDF 368 kb)

Additional file 7: **Figure S6.** Distribution of most abundant taxa across samples. (PDF 405 kb)

Additional file 8: **Figure S7.** Correlation network of vaginal microbiota in the A + N* group. (PDF 202 kb)

Additional file 9: PICRUSt predictive functional profiling (KEGG databases at level 3) between the A–N– and A–N* groups (**Table S3**), the A–N– and A + N+ groups (**Table S4**), the A–N– and A + N* groups (**Table S5**), the A + N– and A + N* groups (**Table S6**), the A + N– and A + N+ (**Table S7)**, the A + N* and A + N+ groups (**Table S8**). **Table S9**: 32 predictive functional categories which differed significantly between any two of the A − N−, A − N*, A + N−, A + N* and A + N+ groups (XLSX 43 kb)

Additional file 10: **Figure S8.** Principal component analysis of 32 predictive functional modules using PICRUSt in level 3 KEGG database. (PDF 324 kb)

Additional file 11: **Figure S9.** Boxplots of 12 predictive functional categories that differed significantly among Nugent score test groups but not between the Amsel criteria groups (*P* < 0.05). (PDF 463 kb)

## Abbreviations

16S rRNA: 16S ribosomal RNA
ANOSIM: analysis of similarity
BV: bacterial vaginosis
NGS: next-generation sequencing
OTU: Operational taxonomic unit
PAST: paleontological statistics
PICRUSt: Phylogenetic investigation of communities by reconstruction of unobserved states
SIMPER: similarity percentage
