# Supplementary material for: Vaginal microbiome variances in sample groups categorized by clinical criteria of bacterial vaginosis
Source: BMC Genomics. 2018 Dec 31;19(Suppl 10):876. doi: 10.1186/s12864-018-5284-7 (PMC6311936; doi:10.1186/s12864-018-5284-7)
Supplement: Supplementary file 1 — Figure S1. Phylum-level microbiota of vaginal samples. (PDF 222 kb) [file 12864_2018_5284_MOESM1_ESM.pdf]

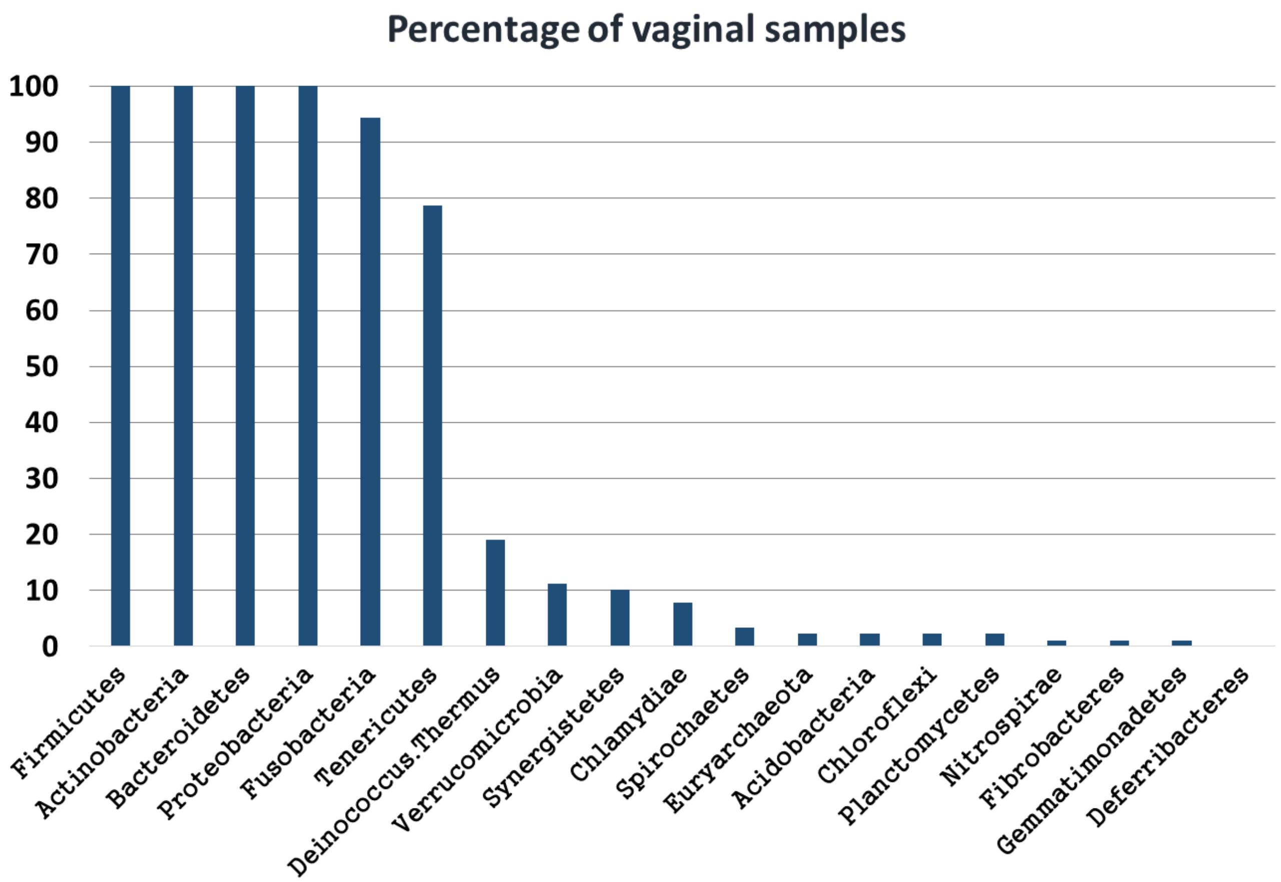

Figure S1. Phylum-level microbiota of vaginal samples. Four phyla, Firmicutes, Actinobacteria, Bacteroidetes, and Proteobacteria, were detectable in all vaginal samples; and two phyla, Fusobacteria and Tenericutes, were present in most of the vaginal samples.
