# Supplementary material for: Vaginal microbiome variances in sample groups categorized by clinical criteria of bacterial vaginosis
Source: BMC Genomics. 2018 Dec 31;19(Suppl 10):876. doi: 10.1186/s12864-018-5284-7 (PMC6311936; doi:10.1186/s12864-018-5284-7)
Supplement: Supplementary file 2 — Table S1. Descriptive statistics of Shannon diversity and richness. Table S2. The p-values for any two groups (PDF 282 kb) [file 12864_2018_5284_MOESM2_ESM.pdf]

Supplementary Tables

Table S1. Descriptive statistics of Shannon diversity index and richness

		Shannon diversity index				*Richness*			
**Groups**	**NO.**	**Min**	**Mean**	**Max**	**SD**	**Min**	**Mean**	**Max**	**SD**
A−N−	19	0.1726 (1)	0.8217 (2)	2.0721 (8)	0.5066	21	50	78	15
A−N*	13	0.6389 (2)	2.0367 (8)	3.2015 (25)	0.7628	29	96	190	46
A+N−	28	0.1344 (1)	0.8473 (2)	1.6554 (5)	0.4091	18	43	73	16
A+N*	5	0.3636 (1)	1.0052 (3)	1.7193 (6)	0.5713	29	48	62	14
A+N+	11	1.5426 (5)	1.8486 (6)	2.0691 (8)	0.1607	24	38	59	11

*The Amsel test: A− means bacterial vaginosis (BV)-negative; A+ means BV-positive
The Nugent score test: N− means BV-negative; N means "intermediate"; N+ means BV-positive
* The numbers in parentheses represent the exponential number of Shannon diversity index.
* Abbreviations: SD, Standard deviation

Table S2. The p-values for any two groups (Wicoxon test)

		p-Values	
Group 1	Group 2	Richness	Shannon index
A−N−	A−N*	1.032E-03	7.691E-05
A−N−	A+N−	1.235E-01	7.556E-01
A−N−	A+N*	8.588E-01	4.056E-01
A−N−	A+N+	2.508E-02	1.834E-05
A−N*	A+N−	9.190E-05	9.103E-06
A−N*	A+N*	2.040E-02	1.937E-02
A−N*	A+N+	4.056E-04	4.585E-01
A+N−	A+N*	4.663E-01	4.783E-01
A+N−	A+N+	3.985E-01	4.773E-09
A+N*	A+N+	1.734E-01	3.205E-03

*The Amsel test: A− means bacterial vaginosis (BV)-negative; A+ means BV-positive
The Nugent score test: N− means BV-negative; N means "intermediate"; N+ means BV-positive
