# Supplementary material for: Vaginal microbiome variances in sample groups categorized by clinical criteria of bacterial vaginosis
Source: BMC Genomics. 2018 Dec 31;19(Suppl 10):876. doi: 10.1186/s12864-018-5284-7 (PMC6311936; doi:10.1186/s12864-018-5284-7)
Supplement: Supplementary file 3 — Figure S2. The Pearson correlation coefficients between richness and Shannon diversity index. (PDF 405 kb) [file 12864_2018_5284_MOESM3_ESM.pdf]

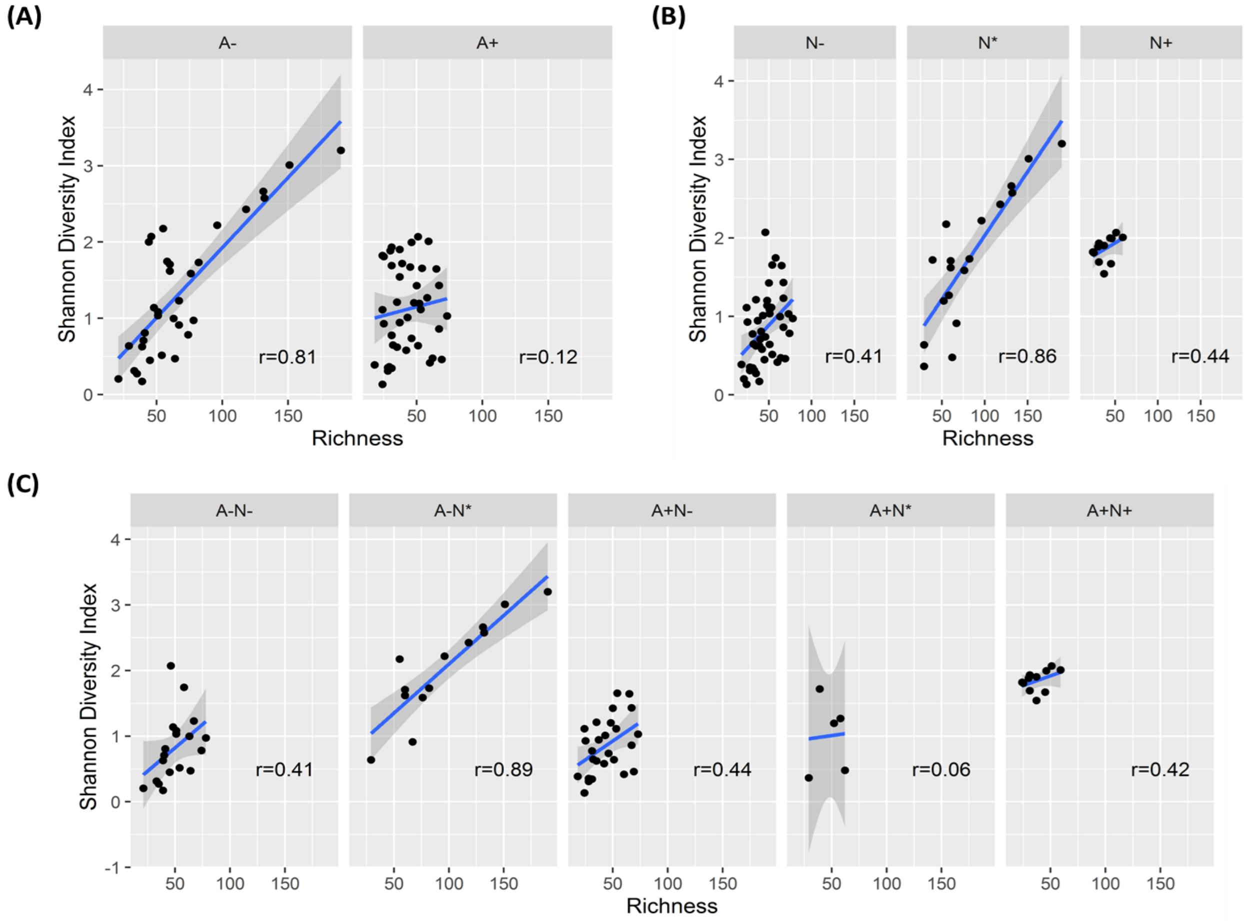

Figure S2. The Pearson correlation coefficients between richness and Shannon diversity index. (A) Two groups according to the Amsel criteria, (B) Three groups according to the Nugent score test, and (C) Five groups according to the two BV tests
