# Supplementary material for: Vaginal microbiome variances in sample groups categorized by clinical criteria of bacterial vaginosis
Source: BMC Genomics. 2018 Dec 31;19(Suppl 10):876. doi: 10.1186/s12864-018-5284-7 (PMC6311936; doi:10.1186/s12864-018-5284-7)
Supplement: Supplementary file 4 — Figure S3. Boxplot of richness and Shannon diversity index in the two subgroups of the A − N− group. (PDF 206 kb) [file 12864_2018_5284_MOESM4_ESM.pdf]

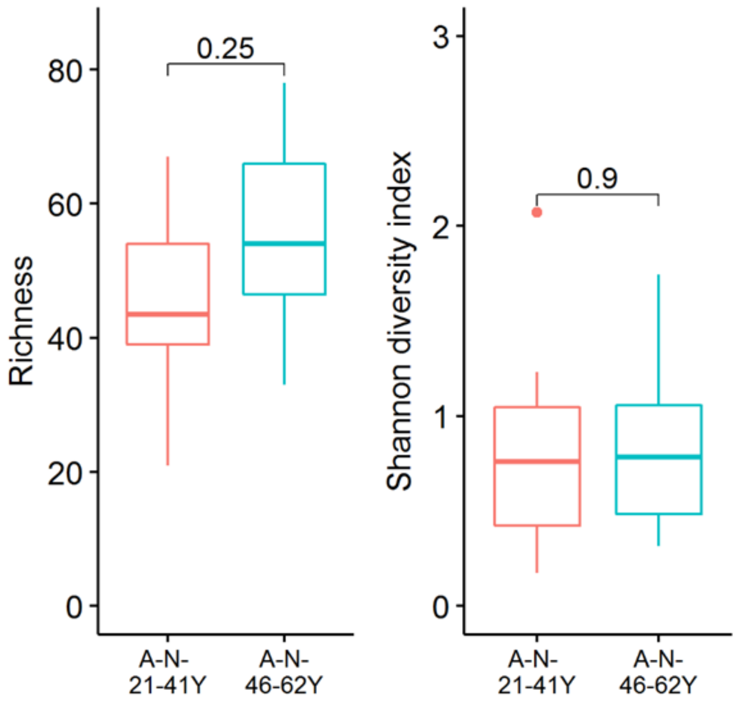


**Figure S3. Boxplot of richness and Shannon diversity index in the two subgroups of the A−N− group.** There was no significant difference in either metric between the subgroups in the A−N− group.
