# Supplementary material for: Vaginal microbiome variances in sample groups categorized by clinical criteria of bacterial vaginosis
Source: BMC Genomics. 2018 Dec 31;19(Suppl 10):876. doi: 10.1186/s12864-018-5284-7 (PMC6311936; doi:10.1186/s12864-018-5284-7)
Supplement: Supplementary file 5 — Figure S4. Principal coordinate analysis (PCoA) plots of vaginal bacterial communities. The PCoA plots were generated by the unweighted UniFrac distance, weighted UniFrac distance, and Bray-Curtis distance. (PDF 426 kb) [file 12864_2018_5284_MOESM5_ESM.pdf]

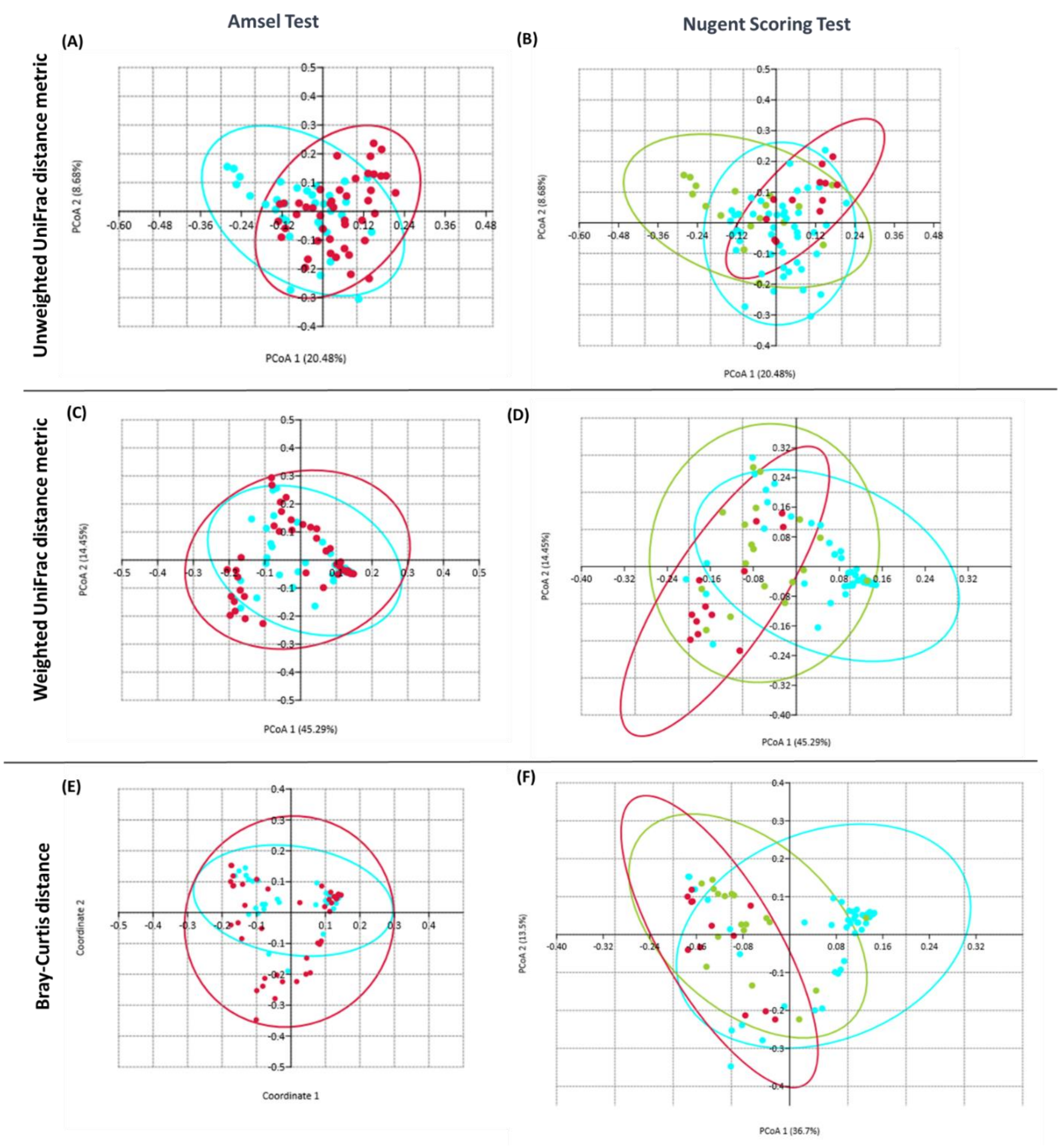

Figure S4. Principal coordinate analysis (PCoA) plots of vaginal bacterial communities.

The PCoA plots were generated by the unweighted UniFrac distance, weighted UniFrac distance, and Bray-Curtis distance. The x- and y-axes are indicated by the first and second coordinates, respectively, and the values in parentheses show the percentages of the community variation. The red dots represent BV-positive women. The blue dots represent BV-negative women. The green dots represent women with "intermedia flora" using the Nugent score test.
