# Supplementary material for: Vaginal microbiome variances in sample groups categorized by clinical criteria of bacterial vaginosis
Source: BMC Genomics. 2018 Dec 31;19(Suppl 10):876. doi: 10.1186/s12864-018-5284-7 (PMC6311936; doi:10.1186/s12864-018-5284-7)
Supplement: Supplementary file 6 — Figure S5. Hierarchical clustering method with Bray-Curtis metric. (PDF 368 kb) [file 12864_2018_5284_MOESM6_ESM.pdf]

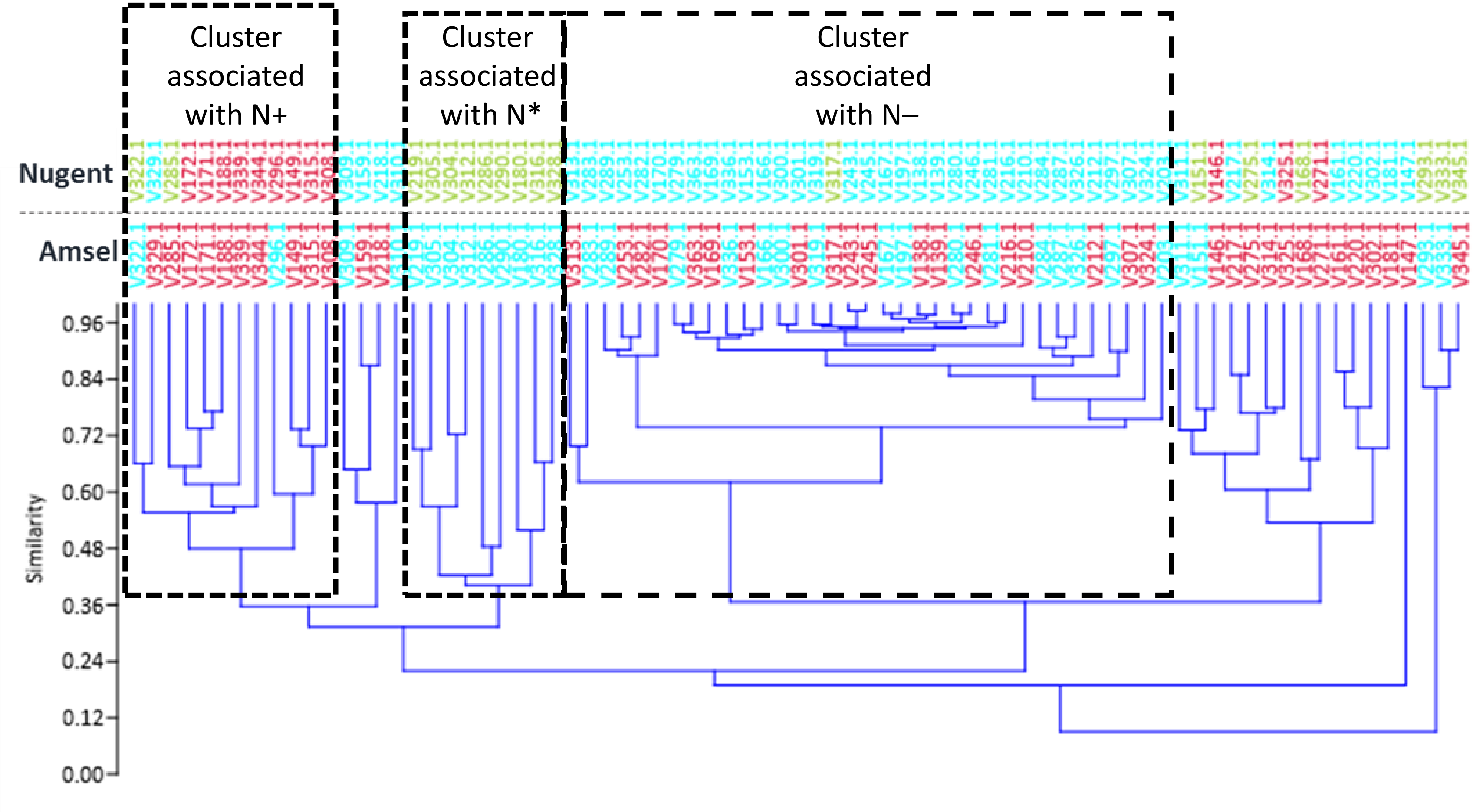

Figure S5. Hierarchical clustering method with Bray-Curtis metric. Most BV-positive women diagnosed with their Nugent scoring test (top row and red ID) clustered at the minimum distance level compared with women diagnosed by the Amsel criteria.
