# Supplementary material for: Vaginal microbiome variances in sample groups categorized by clinical criteria of bacterial vaginosis
Source: BMC Genomics. 2018 Dec 31;19(Suppl 10):876. doi: 10.1186/s12864-018-5284-7 (PMC6311936; doi:10.1186/s12864-018-5284-7)
Supplement: Supplementary file 7 — Figure S6. Distribution of most abundant taxa across samples. (PDF 405 kb) [file 12864_2018_5284_MOESM7_ESM.pdf]

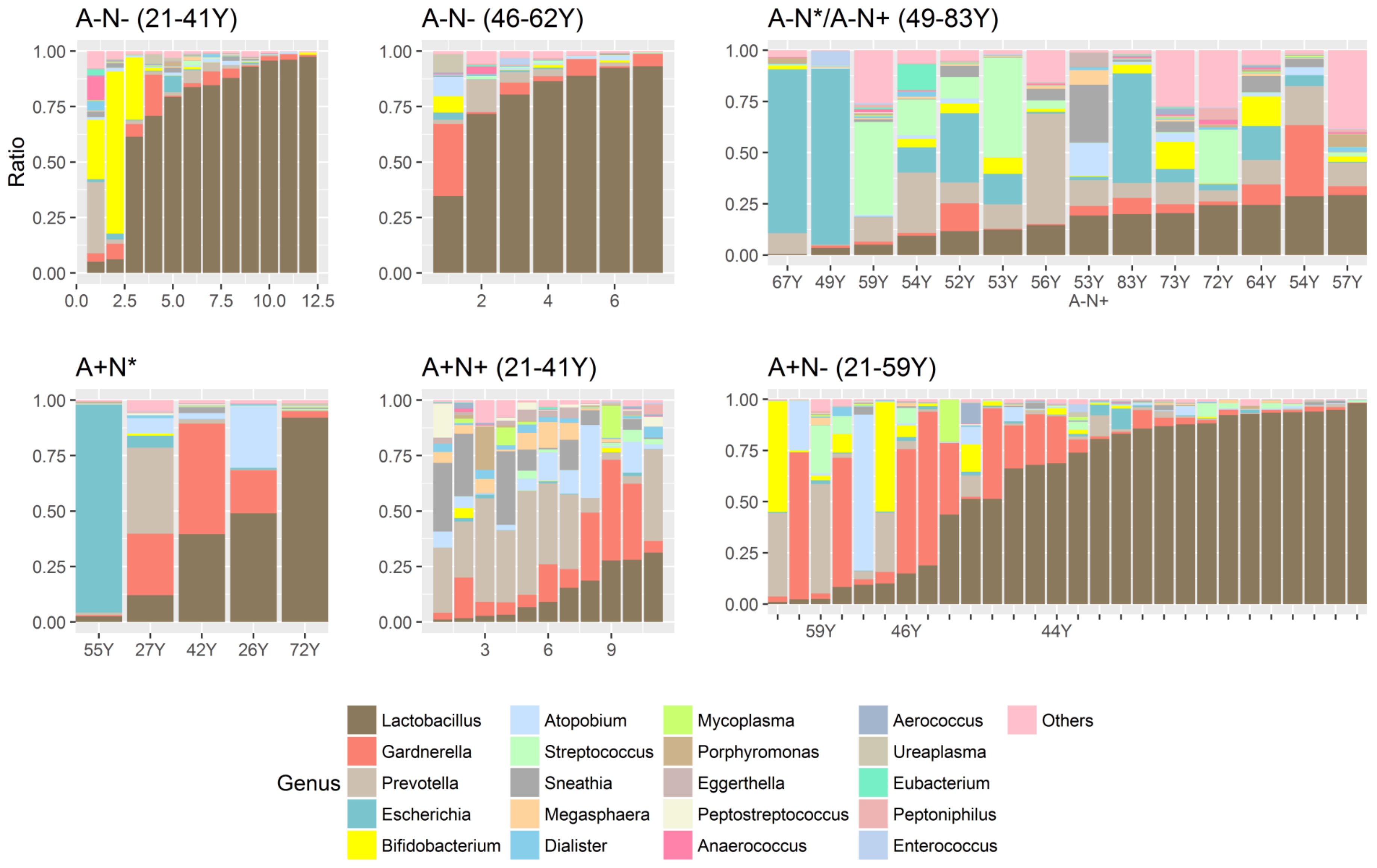

Figure S6. Distribution of the most abundant taxa across samples . Each color represents one bacterial genus, and relative abundances of taxa are represented as bar-charts for each individual sample. There is only one sample in the A−N+ Group.
