# Supplementary material for: Vaginal microbiome variances in sample groups categorized by clinical criteria of bacterial vaginosis
Source: BMC Genomics. 2018 Dec 31;19(Suppl 10):876. doi: 10.1186/s12864-018-5284-7 (PMC6311936; doi:10.1186/s12864-018-5284-7)
Supplement: Supplementary file 8 — Figure S7. Correlation network of vaginal microbiota in the A + N* group. (PDF 202 kb) [file 12864_2018_5284_MOESM8_ESM.pdf]

A+N*

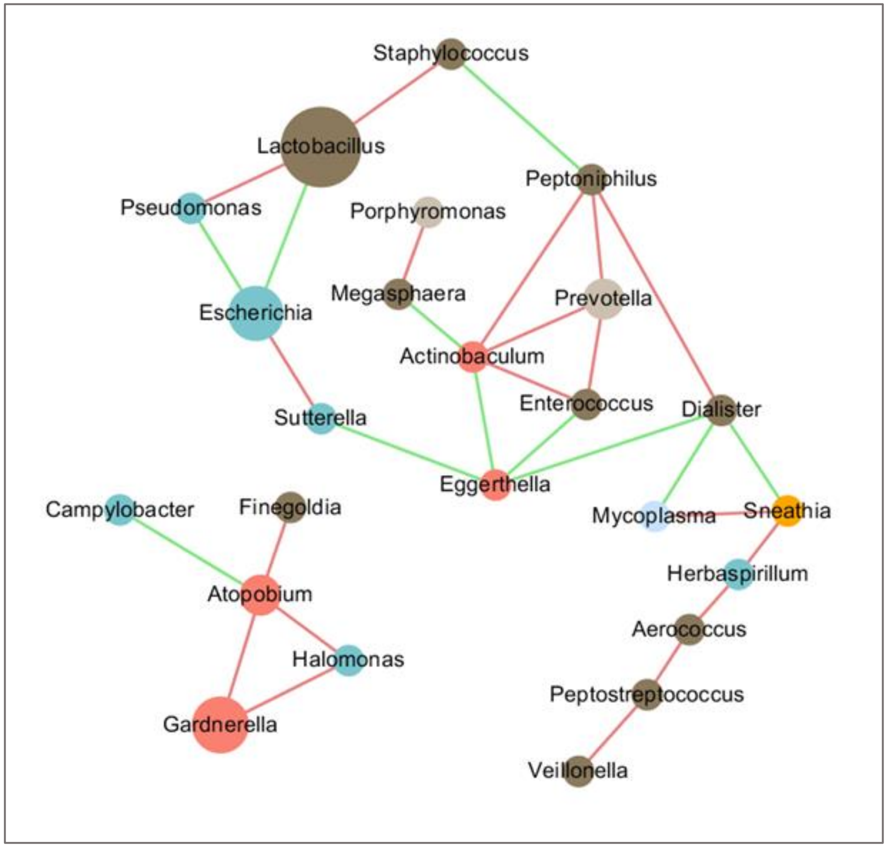

Figure S7 Correlation network of vaginal microbiota in the A+N* group. The figure shows the correlation network using SparCC correlation coefficients at genus level. The nodes represent bacteria genera, and the node size represents the relative abundance. An edge is colored green for a negative correlation and red for a positive correlation.
