# Supplementary material for: Vaginal microbiome variances in sample groups categorized by clinical criteria of bacterial vaginosis
Source: BMC Genomics. 2018 Dec 31;19(Suppl 10):876. doi: 10.1186/s12864-018-5284-7 (PMC6311936; doi:10.1186/s12864-018-5284-7)
Supplement: Supplementary file 10 — Figure S8. Principal component analysis of 32 predictive functional modules using PICRUSt in level 3 KEGG database. (PDF 324 kb) [file 12864_2018_5284_MOESM10_ESM.pdf]

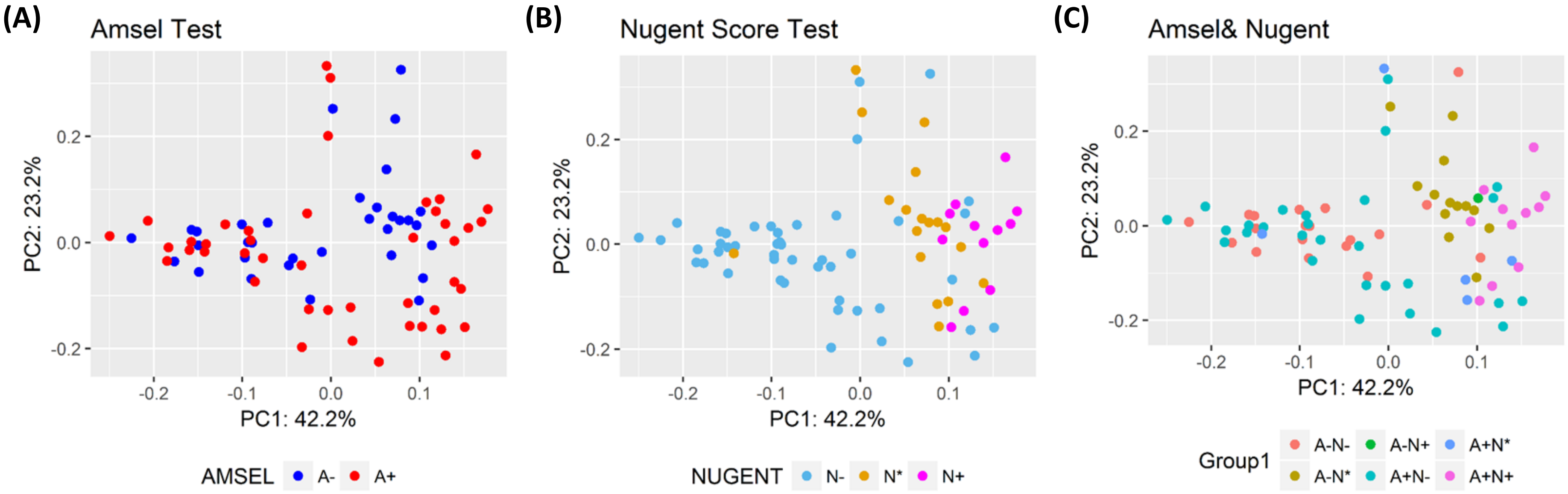


**Figure S8. Principal component analysis of 32 predictive functional modules using PICRUSt in level 3 KEGG database**. Each vaginal subject is represented by a colored point. (A) Red indicates A+ subjects, and blue indicates A− subjects. (B) The three colors denote the three classes defined by the Nugent score test. Blue indicates N− subjects, orange N* subjects, and pink N+ subjects. The vaginal subjects are separated into two clusters: the first consisting mostly of N− subjects, and the second of N* and N+ subjects. (C) The six colors denote the six groups defined by the two BV tests.
