# Supplementary material for: Vaginal microbiome variances in sample groups categorized by clinical criteria of bacterial vaginosis
Source: BMC Genomics. 2018 Dec 31;19(Suppl 10):876. doi: 10.1186/s12864-018-5284-7 (PMC6311936; doi:10.1186/s12864-018-5284-7)
Supplement: Supplementary file 11 — Figure S9. Boxplots of 12 predictive functional categories that differed significantly among Nugent score test groups but not between the Amsel criteria groups (P < 0.05). (PDF 463 kb) [file 12864_2018_5284_MOESM11_ESM.pdf]

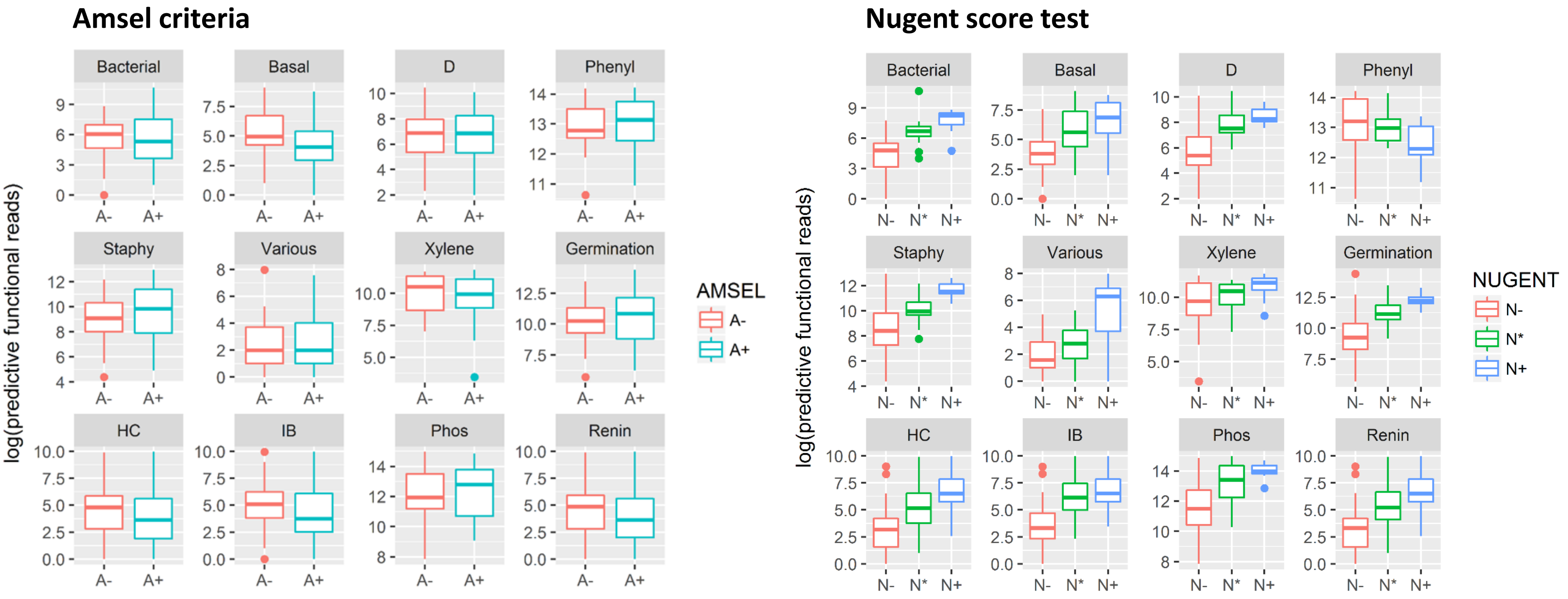

Figure S9. Boxplots of 12 predictive functional categories that differed significantly among Nugent score test groups but not between the Amsel criteria groups (*P* < 0.05). The full module names are abbreviated as follows (1) Bacterial = Bacterial invasion of epithelial cells; (2) Basal = Basal transcription factors; (3) D = D-Arginine and D-ornithine metabolism; (4) Phenyl = Phenylpropanoid biosynthesis; (5) Staphy = Staphylococcus aureus infection; (6) Various =Various types of N glycan biosynthesis ; (7) Xylene = Xylene degradation; (8) Germination = Germination; (9) HC = Hypertrophic cardiomyopathy HCM; (10) IB =Isoflavonoid biosynthesis; (11) Phos = Phosphotransferase system PTS; (12) Renin =Renin angiotensin system.
